# Androgenetic Alopecia: Quality-of-life and Associated Lifestyle Patterns

**DOI:** 10.4103/0974-7753.77510

**Published:** 2010

**Authors:** Neena Sawant, Siddhi Chikhalkar, Varun Mehta, Malvika Ravi, Bhushan Madke, Uday Khopkar

**Affiliations:** Department of Psychiatry, Seth GS Medical College and KEM Hospital, Mumbai, Maharashtra, India; 1 Department of Skin & VD, Seth GS Medical College and KEM Hospital, Mumbai, Maharashtra, India

**Keywords:** Alopecia Areata, histopathology, non-scarring alopecia

## Abstract

**Background:**

Androgenetic alopecia (AGA) is a condition, which is an important psychosocial problem. The hormonal variations causing AGA are known, but whether behavioral patterns and lifestyle influence the condition and which age groups they influence is uncertain and such factors have not been studied in detail.

**Objectives:**

To compare association of lifestyle patterns with androgenetic alopecia, prevalence of psychiatric symptoms and resulting quality-of-life (QoL) between two age groups of males with AGA.

**Materials and Methods:**

Male subjects in each of the two age groups attending the hair clinic diagnosed with AGA were administered a questionnaire on lifestyle patterns. HAIRDEX and symptom checklist-90 (SCL-90) to study the presence of psychosocial problems and QoL were used. The stress experienced by such patients was studied by a stressful life events scale.

**Results:**

Of the 37 patients studied, 23 were in younger age group (average age) and 14 were in the older age group (average age). No significant difference was found in lifestyle, as far as eating habits, physical activity, occupational activity and leisure time were concerned. However, the younger age group had a significantly better psychological health. (*P*=0.013). On assessing the QoL, self-assurance seemed better in younger age group (*P*=0.014), reflecting changing societal trends, causing better acceptance of hair loss. On the other subscales, emotions seemed to be more affected in the younger age group, while older patients had worse functioning, more symptoms and more sense of stigmatization. On assessing SCL-90, no significant psychopathological difference was found between both the groups; however the older patients appeared to have more psychological symptoms on almost all scales scoring highly on obsessive–compulsive, interpersonal sensitivity and depression subscales. No significant difference in stressful life events at the time of onset of alopecia was noticed although older patients scored higher on this scale. Family history was found to be significantly associated with the onset of alopecia (*P*=0.0448).

**Conclusions:**

We concluded that lifestyle factors and stressful life events are not significantly affected by the onset of AGA. Only heredity seems to be associated with hair loss. Quality-of-life is affected in both the age groups but younger patients seem to have better self-assurance as well as better psychological health.

## INTRODUCTION

Hair loss is a universal problem. Androgenetic alopecia (AGA) more commonly known as male pattern baldness affects up to 50% of men worldwide.[1] The disorder occurs in almost all patients before 40 years and in many patients below the age of 30 years.[2] A little is known about the etiology of AGA. Most studies looking into the cause of AGA correlate this disease with hormonal variations especially dehydroepiandrosterone (DHEA) levels.[3] Considering how lifestyle factors influence hormonal levels greatly, it could be presumed that lifestyle and behavioral patterns may contribute to the occurrence and severity of AGA. This view is supported by a study done by Alfonso *et al*. in 2005[4] in male subjects with AGA, where a majority of them reported that hair loss affected their personal attractiveness and social life. Low self-esteem and loss of self-confidence have also been reported by Williamson *et al*. in a study in 2001.[5]

Additionally, hair loss has been known to cause significant distress in men. A study done by Cash *et al*. in both sexes found AGA to be a stressful condition affecting the psychological functioning of an individual.[6]

A study done by Cash *et al*. found a greater degree of psychological impact of hair loss among younger men and those with earlier onset hair loss.[7] Since adolescents and young adults meet with the challenge of fitting in with their peers, an additional stress of hair loss could be a burden to their coping abilities. Thus to ascertain the degree of psychological adversity we decided to compare two age groups of males with AGA. The subjects in both groups were then compared for psychopathology, stressful life events, association of lifestyle patterns with disease and quality-of-life (QoL) after onset of hair loss.

## MATERIALS AND METHODS

### Study site

The study was carried out in the weekly ‘hair clinic’ of the dermatology outpatient department of a tertiary care municipal hospital in Mumbai.

### Study subjects

The male subjects selected for the study had been diagnosed as suffering from AGA by dermatologists in the clinic on the basis of clinical criteria, including pattern of hair loss. Forty five consecutive male patients attending the hair clinic were selected; out of which two refused to give consent and six patients were excluded as they were not fulfilling the inclusion criteria.

### Ethics

After obtaining permission from the institutional ethics committee, the male subjects satisfying the inclusion and exclusion criteria were recruited for the study.

### Inclusion criteria

Male patients presenting with AGA having grade IIa to VII (according to Hamilton–Norwood classification of hair loss) between the age of 15 and 60 years were recruited for the study. They were divided into two age groups on the basis of age.

Group A: 15 to 25 years (both ages inclusive)

Group B: 26 to 50 years (both ages inclusive)

### Exclusion criteria

Patients with existing psychopathology and any other associated pre-existing medical illness were excluded from the study.

### Tools

1. Lifestyle indices: The questionnaire used for lifestyle indices was prepared and validated by the dermatologists with subscales on eating habits, occupation, leisure and recreation, physical health and psychological health and addictions. Each subscale had a set of questions, each rated on a five-point Likert rating.
2. HAIRDEX: HAIRDEX is an instrument developed to measure QoL in patients with disorders of the hair and scalp. The scale included questions under five categories: a) Emotions; b) Functioning; c) Symptoms; d) Self-assurance and e) Stigmatization. The replies were graded from 0 to 4 by subjects according to frequency with which they occur.
3. Symptom Check List-90-R (*SCL-90-R*) outpatient psychiatric rating scale: This is a scale consisting of 90 questions to evaluate psychopathology. The respondents reported how much discomfort they experienced over the past week and past day with selected symptoms/psychological states. Responses were scored on a 0 to 4 continuum (0=not at all, 4=extremely).The SCL-90-R covers nine symptom dimensions, which are (1) somatization (perceptions of bodily dysfunction); (2) obsessive–compulsive; (3) interpersonal sensitivity (feelings of personal inadequacy or inferiority); (4) depression; (5) anxiety; (6) hostility; (7) phobic anxiety; (8) paranoid ideation and (9) psychoticism.
4. A stressful life events scale was prepared to assess the stressful life events experienced by the individual during the period of hair loss was first noticed. It includes various stressful life events grouped under financial, family-related, social and personal stressors. The scale was rated with a ‘yes’ response (graded as 1 mark) or a ‘no’ (graded as 0 mark).

### Methods

A proforma was prepared to collect information on the age of onset, duration of alopecia, demographic variables and details of family history. All the subjects were administered scales by the investigators to gather information on lifestyle indices, QoL, psychopathology and stressful life events.

### Statistical analysis

The data from both groups was analyzed with respect to psychiatric symptoms, lifestyle indices and QoL by means of *t*-test and Fischer’s exact test where applicable.

## RESULTS

Of the 37 patients who were recruited, 23 (62%) were between the age of 15 and 25 (group A) and the remaining 14 (38%) were included in the older age group (B). In both groups interviewed, about 60–70% were graduates, about 70% belonged to a middle class family (Kuppuswami’s grading) and 35% of those in group B were married. Family history of alopecia was seen in 65% patients of group A and 28% of group B, which was statistically significant. Seborrheic dermatitis was seen in 42% of patients in group B and 56% patients in group A.

When lifestyle factors [Table 1] were analyzed, the mean score of patients in group A was higher than group B. The differences were statistically significant in the subscale of psychological health and addictions (*t*=3.8 and *P*=0.013).

**Table 1:** Lifestyle factors and age of presentation

Lifestyle factors	Age of presentation					
	Group A		Group B		*t*	*P*
	Mean	SD	Mean	SD		
Eating habits	13.69	3.95	11.92	3.43	1.382	0.175
Physical	4.91	1.70	4.64	2.30	0.408	0.685
Occupational	10.43	3.51	9.57	4.05	0.684	0.49
Leisure	4.78	2.71	4.28	2.89	0.527	0.6
Psychological and addictions	2.0	3.13	1.7	3.8	2.60	0.013

The QoL subscales [Table 2] results showed the younger age group (group A) to be less affected as far as stigmatization, functioning, self-assurance and symptoms subscales were considered. They fared poorly with higher scores than older patients as far as emotions were concerned. However, the younger age group proved to be statistically more self-assured (*t*=1.852, *P*=0.072).

**Table 2:** Age of presentation and HAIRDEX score

QoL subscales	Age of presentation					
	Group A		Group B		*t*	*P*
	Mean	SD	Mean	SD		
Emotions	25.26	13.14	20.92	16.21	0.890	0.379
Functioning	9.60	10.91	11.85	7.55	0.676	0.5
Symptoms	6.91	4.89	9.28	6.71	01.24	0.223
Self-assurance	10.52	5.05	14.28	7.32	1.852	0.072
Stigmatization	13.17	9.55	13.9	12.2	0.209	0.835

When both the groups were compared for psychopathology on the various symptom- constructs of symptom check list 90 [Table 3], no significant difference was seen in subscales of somatization, obsession–compulsion, interpersonal sensitivity, depression, anxiety, anger-hostility, phobic anxiety, paranoid ideation and psychoticism. Older people seemed to be having more neuroticism, such as obsessive–compulsive symptoms and depression, as compared to the younger age group who scored higher in the anger and hostility subscale.

**Table 3:** Age of presentation as related to score on SCL-90 subscales

SCL-90 subscales	Age of presentation					
	Group A		Group B		*t*	*P*
	Mean	SD	Mean	SD		
Somatization	3.17	5.11	3.57	4.23	0.2440	0.808
Obsessive–compulsive	7.39	7.14	9.21	10.94	0.596	0.55
Interpersonal sensitivity	4.174	6.08	4.21	3.18	0.0171	0.98
Depression	4.913	8.37	5.57	9.37	0.221	0.825
Anxiety	1.43	2.39	1.9	7.21	0.304	0.762
Anger and hostility	2.913	4.814	2.78	4.42	0.0803	0.936
Phobic anxiety	0.39	1.07	0.92	2.55	0.892	0.378
Paranoid ideation	2.13	4.49	1.92	3.24	0.146	0.884
Psychoticism	0.565	1.12	0.92	347	0.40668	0.643

A comparison of differences in the stressful life events [Table 4], considered in four life-event categories of finances and other achievements, personal, social and familial stressors since the onset of hair loss revealed no statistically significant difference between both age groups; the mean number of events for group A being 2.34 and group B being 3.14 (*P* 0.4776).

**Table 4:** Age of presentation and stressful events

Stressors	Age of presentation					
	Group A		Group B		*t*	*P*
	Mean	SD	Mean	SD		
Total	2.347	2.963	3.142	3.416	0.7471	0.460
Familial	0.434	0.727	0.928	1.207	1.558	0.128
Involving finances and achievements	1.260	1.657	1.357	1.737	0.168	0.867
Personal stressors	0.478	0.845	0.714	1.139	0.168	0.867
Social stressors	0.173	0.491	0.142	0.363	0.721	0.690

On analyzing association of severity of hair loss with QoL [Table 5], patients with more visible hair loss reported higher mean scores (i.e. they were more afflicted) on subscales of emotions, functioning, self-assurance and stigmatization.

**Table 5:** Visibility of hair loss in correlation with QoL subscales

QoL Subscales	Hair loss visibility					
	More visible		Less visible		*t*	*P*
	Mean	SD	Mean	SD		
Emotions	24.053	15.24	23.16	13.7	0.1871	0.665
Functioning	12.31	12.61	11	13.19	0.306	0.84
Symptoms	773	5.53	789	5.99	0.084	0.933
Self-assurance	10.26	5.03	8.83	5.40	0.833	0.41
Stigmatization	13.94	11.34	12.99	9.77	0.2723	0.787

On correlating presence of family history with age of onset of hair loss using Fischer’s exact test [Table 6], a statistically significant association of presence of hair loss in a male sibling or parent was seen with early-onset of hair loss (two-sided *P* value=0.0448).

**Table 6:** Presence of family history at age of presentation

Family history of AGA	Age of presentation			
	Group A	Group B	Total	*P* value
Present	15 (78)	4 (22)	19	0.0448
Absent	8 (44)	10 (56)	18	
Total	23	14	37	

Figures in parentheses are in percentage

## DISCUSSION

Very few studies have associated multiple lifestyle factors with AGA. An example would be an observational study by Trüeb *et al*. which links to smoking and hair loss.[8] In our study, patients of the younger age group reported an overall healthier lifestyle as compared to the older age group in all five parameters assessed, viz. eating habits, physical activity, occupation, leisure and recreation, emotional health and addictions. Thus, the study discounts any causative role of lifestyle patterns in the onset of hair loss. In fact, a statistically significant difference was found between both groups regarding emotional health and absence of addictions. The better scores of younger patients indicate that they had a more positive outlook towards life, a better ability to get along with peers, better sleeping habits (*t*=2.60, *P*=0.013). This may be explained by the fact that there is a relatively lesser time since onset of hair loss. It could be postulated that chronicity of the condition may add to psychological tolls of hair loss.

Quality-of-life due to hair loss was assessed by administration of the HAIRDEX under five subscales: emotions, functioning, self-assurance, stigmatization and symptoms.[9] We found that the group of older patients seemed to be more severely afflicted as far as the subscales of functioning, stigmatization and symptoms were concerned.

The reason for the better functioning and less stigmatization in the younger patient group is the increasing influence of fashion and media, which now portrays baldness as a new trend with younger people adopting newer hairstyles as per their role models. Today’s culture being metro-sexual has definitely helped these younger patients suffering from AGA. The younger patients only fared worse in the subscale entitled ‘emotions’. Coupled with the knowledge that an inordinate importance is given to personal appearance in the younger age group covered by our study (15 to 25 years), our findings are in direct contrast to an older study by Wells (1995), which notices more marked loss of self-esteem and feelings of looking unattractive in younger men.[10] This could be due to changing attitudes towards baldness in the direction of increased social acceptance. A study carried out using the same scale on female patients, revealed impaired QoL (i.e. higher scores) in patients with varying degrees of hair loss, even in those whose hair loss is not clearly visible.[11] Patients experience emotions of annoyance, frustration and humiliation. Also, social life and interaction, especially with the opposite sex is affected.[9] A study by Alfonso *et al*. reports that major concerns faced include losing a part of personal attractiveness, becoming bald, getting older, negative effects on social life and feelings of depression.[4] A change noticed in both groups of patients is the feeling of ‘stigmatization’ which includes ‘feeling like an outsider’ and being laughed at by others.[9] These feelings could be due to perception of peer pressure exaggerating the need for social acceptance.

Interestingly, a statistically significant better self-assurance scores were found among the younger patient group (*P*=0.014). The presence of better self-assurance is undoubtedly the result of a generational attitudinal difference between younger and older patients of AGA. Again it correlates with our previously mentioned finding of better psychological health and lifestyle in younger patients.

A study on AGA showed that men whose fathers had hair loss were 2.5 times as likely to have had some level of hair loss compared to men whose fathers had no hair loss.[12] Our study reports a similar finding when presence of early-onset hair loss in first-degree male relatives of the patients was studied. The results of Chi-square test showed a statistically significant difference such that patients of a younger age group were more likely to have a male relative suffering from early-onset AGA. Thus, heredity can be said to be associated with age of onset of hair loss.

To date there is very little data to find a temporal relationship between the degree of hair loss and psychosocial variables associated with AGA. A study done by Wells *et al*. reports that increasing degrees of hair loss were associated with loss of self-esteem, depression, introversion, neuroticism and feeling unattractive.[10] These effects were more marked for young men in the case of self-esteem, introversion and feeling unattractive.[8] Interestingly, the results of two studies done by van der Donk *et al*. negates the view of psychological impairment accompanying AGA although QoL does cause psychosocial problems.[13,14] In our study when SCL-90[15] (the symptom check list) outpatient psychiatric rating scale was administered to both the groups, there wasn’t any significant impairment in psychological functioning in both the age groups. However, symptoms of depression and obsessive–compulsive disorder was found to be more in group B as compared to group A, which can be correlated with our findings of greater impairments in QoL in the same group. Patients in both the age groups scored highest on obsessive–compulsive, depression and interpersonal sensitivity scales. An increase in sensitivity regarding interpersonal relations is supported in a study carried out by Franzoi *et al*.,[16] where men with AGA had more public self-consciousness and considered themselves less attractive. Also they could not find any evidence that the onset of hair loss caused changes in level of public self-consciousness, supporting our view that interpersonal sensitivity was uniformly increased in both the groups.

The contribution of stress to the onset of AGA has not been studied in detail. A study by Brajac in 2003, who attempted to link alopecia areata with stressful life events, was unable to establish causal relationship.[17] Occurrences of 51 life events commonly associated with stress in patients at around the time of onset of hair loss were looked into. Patients belonging to older age group reported a higher average number of events (3.14); however, no statistically significant difference was found. Thus, we can see no association between stress and age of onset of AGA.

The degree of visibility of hair loss might affect the QoL of patients with male pattern baldness. Therefore, a further analysis was done, classifying the patients into two groups based on their current Norwood–Hamilton stage of AGA, those with less visible hair loss (stage IIa to III) and more visible hair loss (stage IIIa to IV). When their scores on QoL were compared by the *t*-test, it was noticed that patients with more visible hair loss scored higher on the emotions, self-assurance, functioning and stigmatization subscales. This can be correlated with the perception of visible balding by the peer group. A similar study on female patients of AGA, with the use of the same scale, showed that patients with more visible hair loss scored higher on all five subscales.[11]

## CONCLUSIONS

Family history definitely seems to play a role and appears to be associated with age of onset of hair loss and patients with positive family history seem to present at young age. While overall lifestyle patterns seem to have no effect on AGA, psychological health seems to be useful in coping with AGA. Younger patients seem to be showing improved functioning, self-assurance and decreased stigmatized feelings. Stressful events while higher in older patients seem to have no bearing on age of presentation of alopecia androgenetica. While AGA patients score higher on neuroticism as psychopathology, no significant psychopathological effects of AGA have been noted. Younger patients seem to retain QoL better in spite of AGA; QoL seems to be affected by visibility of hair loss.
